# Case Report: Rare pheochromocytoma in a patient with Li–Fraumeni syndrome: a 3-event, 4-hit model of pathogenesis

**DOI:** 10.3389/fonc.2026.1714565

**Published:** 2026-03-11

**Authors:** Yi Liu, Aaron Dinerman, Ina Lee, Liqiang Xi, Manoj Tyagi, Naris Nilubol, Ashley Grossman, Payal P. Khincha, Kenneth Aldape, Kathleen Calzone, Karel Pacak, Mark Raffeld

**Affiliations:** 1 Genetics Branch, Center for Cancer Research, National Cancer Institute, National Institutes of Health, Bethesda, MD, United States; 2 Surgery Branch, Center for Cancer Research, National Cancer Institute, National Institutes of Health, Bethesda, MD, United States; 3 Laboratory of Pathology, Center for Cancer Research, National Cancer Institute, National Institutes of Health, Bethesda, MD, United States; 4 Surgical Oncology Program, Center for Cancer Research, National Cancer Institute, National Institutes of Health, Bethesda, MD, United States; 5 Neuroendocrine Tumor Unit, Royal Free Hospital, London, and Green Templeton College, University of Oxford, Oxford, United Kingdom; 6 Clinical Genetics Branch, Division of Cancer Epidemiology and Genetics, National Cancer Institute, National Institutes of Health, Bethesda, MD, United States; 7 Section of Medical Neuroendocrinology, National Institute of Child Health and Human Development, National Institutes of Health, Bethesda, MD, United States

**Keywords:** Li-Fraumeni syndrome, molecular pathogenesis, NF1, pheochromocytoma, TP53

## Abstract

Li–Fraumeni syndrome (LFS) is a rare autosomal dominant hereditary cancer predisposition syndrome caused by germline *TP53* pathogenic variants. Despite numerous studies of associated cancers with this syndrome, cases of pheochromocytoma have not been well documented. We present a patient from an LFS family who developed a right adrenal mass with a clinical presentation consistent with a pheochromocytoma. Genetic studies of this tumor identified a germline *TP53* pathogenic variant (c.818G>A; p.Arg273His) with somatic loss of the wild-type allele (loss of heterozygosity, LOH). In addition, a likely somatic *NF1* pathogenic variant was found with concomitant LOH. There were no reported cases of pheochromocytoma in the family history. In addition, several bile duct adenomas (BDAs) were discovered and biopsied intraoperatively. Sequence analysis of one BDA revealed a likely somatic *FGFR2::FKR* pathogenic fusion and the identical germline *TP53* pathogenic variant. In contrast to the pheochromocytoma, the BDA showed no evidence of a second *TP53* alteration that might suggest that *TP53* had played a role in its pathogenesis. This case highlights the rare presentation of pheochromocytoma in LFS and provides a molecular hypothesis of how this tumor may have developed.

## Introduction

Li–Fraumeni syndrome (LFS) is an autosomal dominant cancer predisposition syndrome, primarily caused by germline *TP53* pathogenic or likely pathogenic (P/LP) variants (1). The classical criteria for clinical diagnosis include the occurrence of sarcoma prior to the age of 45 in the affected individual, the presence of a first-degree relative with cancer diagnosed under age 45, and identification of a similar-lineage family member with any cancer diagnosed under age 45 or sarcoma at any age (1). LFS is characterized by a heightened incidence of both pediatric and adult malignancies (2). Affected families commonly exhibit a diverse spectrum of cancer types that include osteosarcoma, soft tissue sarcoma, acute leukemia, breast cancer, brain cancer, and adrenocortical tumors (3). In addition, there is an elevated susceptibility to many other cancer types, including hematological malignancies, melanoma, Wilms’ tumor, and cancers of the stomach, colon, pancreas, esophagus, lung, and gonadal germ cells, among others (2, 4). LFS presents with an early onset of cancer; the risk for women is 41% by the age of 30, and for men, it is 47% by the age of 45 (5).

Pheochromocytomas represent neuroendocrine tumors derived from chromaffin cells of the adrenal medulla. These tumors can exhibit biochemical activity as well as recurrent/metastatic potential (6). Approximately 40% of cases are characterized by the presence of germline P/LP variants in a variety of genes that include *NF1*, *RET*, *SDH* subunits *A–D*, *SDHAF2*, *VHL*, *MAX*, *TMEM-127*, *EPAS1*, and others (7, 8). However, despite this high degree of heritability, the association of pheochromocytoma with biallelic germline *TP53* and somatic P/LP variants has not been previously reported in the literature.

This report describes a case of an individual with a germline *TP53* pathogenic variant whose initial malignancy manifested as a biochemically active pheochromocytoma. Next-generation sequencing (NGS) analysis of this tumor detected the patient’s known pathogenic germline *TP53* variant accompanied by somatic loss of heterozygosity (LOH) and a somatic pathogenic variant in *NF1*, a frequently mutated gene in both familial and sporadic pheochromocytoma, with concomitant LOH. Several bile duct adenomas were also found during the index operation, for which NGS analysis identified the germline *TP53* pathogenic variant without evidence of a pathogenic alteration on the alternate allele, and an *FGFR2::FRK* fusion.

## Results

### Clinical findings

The patient is a white male in his 30s who was diagnosed with LFS based on family history and found to have a germline pathogenic variant in *TP53* (c.818G>A; p.Arg273His), with no prior personal cancer history. He has been actively participating in a natural history study of LFS at the National Institutes of Health (NIH) since the age of 25. The study protocol included annual whole-body MRI imaging that was unremarkable except for the presence of an indolent brain lesion thought to be a benign choroid plexus tumor. On surveillance whole-body MRI done at age 37, the patient was found to have an enlarging right adrenal mass and clinical manifestations including night sweats and intermittent daily palpitations that were suspicious for a biochemical profile consistent with pheochromocytoma (subsequent preoperative CT, **Supplementary Figure S1**). Subsequent laboratory analysis revealed elevated plasma-free normetanephrine and metanephrine levels, measured by liquid chromatography–tandem mass spectrometry (LC-MS/MS) at Mayo Clinic Laboratories. Plasma normetanephrine was at 10 nmol/L (normal < 0.90 nmol/L), and plasma metanephrine was 0.98 nmol/L (normal < 0.50 nmol/L). The case was discussed in a multidisciplinary tumor board with an ultimate consensus on operative intervention to remove the right adrenal mass. Because of the retrocaval extension of the tumor, a midline upper laparotomy and right adrenalectomy were performed, with en bloc resection of the bilobed retrocaval mass. During the surgery, three incidental small subcentimeter lesions were noted on the surface of the liver and excised for pathologic examination due to a concern for liver metastasis. The patient underwent appropriate adrenoreceptor blockade preoperatively and experienced an uncomplicated postoperative course with discharge after a 3-day hospital stay. Both specimens (retrocaval mass and one of the superficial liver lesions) were sampled for tumor/normal paired whole exome sequencing (TNWES).

### Histopathology

**Figure 1:**
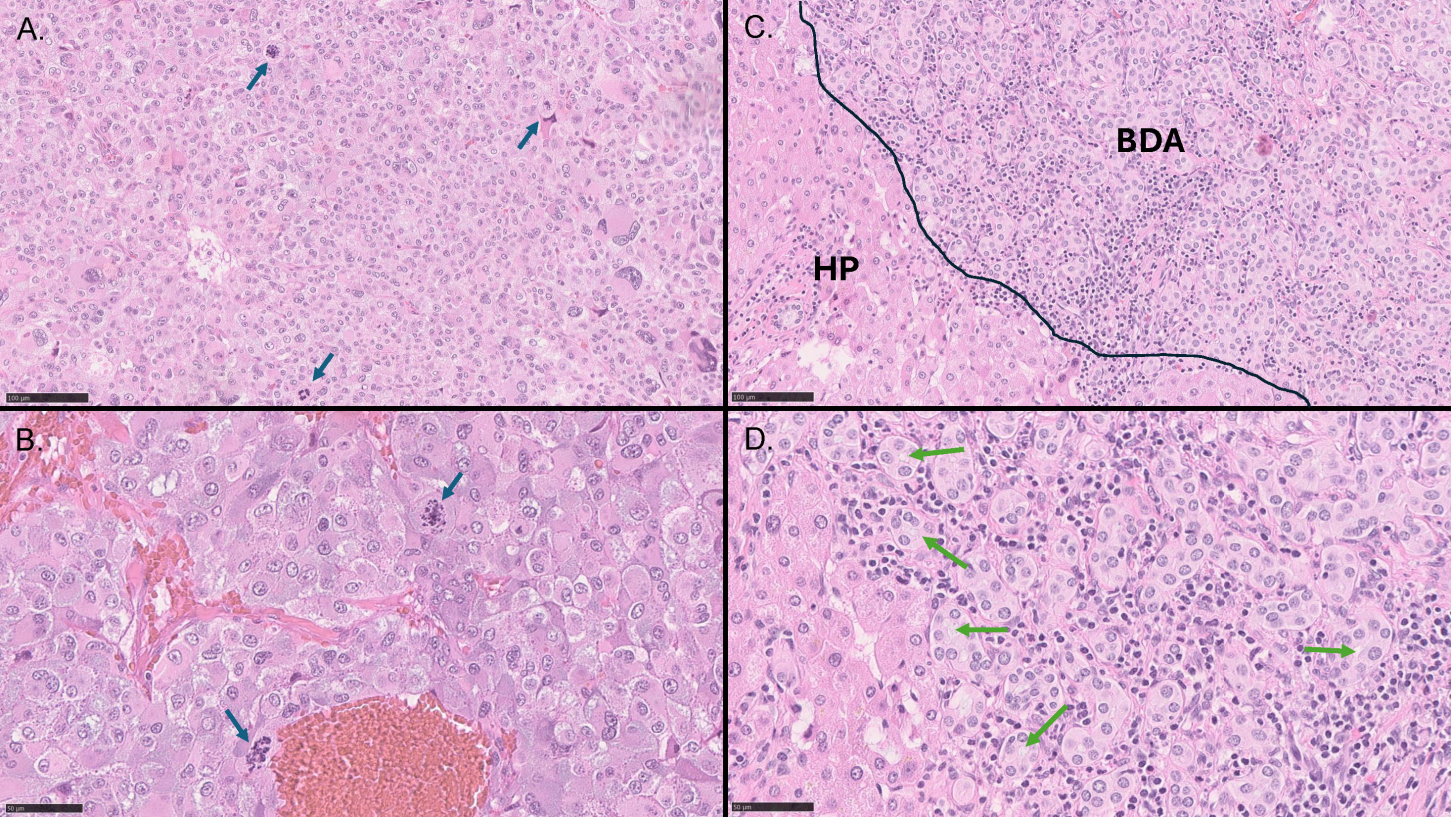
Histology of pheochromocytoma and bile duct adenoma. **(A, B)** Representative photomicrographs of pheochromocytoma [hematoxylin and eosin stain, low **(A)** and high magnification **(B)**] demonstrating sheets and nests of large tumor cells with abundant amphophilic cytoplasm, nuclear pleomorphism with atypical giant cells, and atypical mitoses (blue arrows). **(C, D)** Photomicrographs of bile duct adenoma. **(C)** Bile duct adenoma with uniformly distributed small duct-like structures (BDA) separated by a lymphocytic infiltrate and adjacent hepatic parenchyma (HP). **(D)** Higher magnification illustrating duct-like structures with central lumina (green arrows). Black bars are internal size markers [**(A, C)** = 100 μm, **(B, D)** = 50 μm].

Histological examination of the right adrenal tumor revealed a tumor with a diffuse growth pattern, composed of large cells with nuclear pleomorphism, and a relatively high mitotic index of 4 per 10 high-power fields, consistent with pheochromocytoma (**Figures 1A, B**). A singular focus of vascular invasion was also observed (not shown). Evaluation based on the *Pheochromocytoma of the Adrenal Gland Scoring Scale* (PASS) yielded a score of 7, indicative of an exceedingly aggressive tumor (9). The superficial liver lesions were diagnosed as bile duct adenomas characterized by a proliferation of small tubular structures lined by bland cuboidal cells without atypia or mitotic activity (**Figures 1C, D**).

### *TP53* germline variant and family history

**Figure 2:**
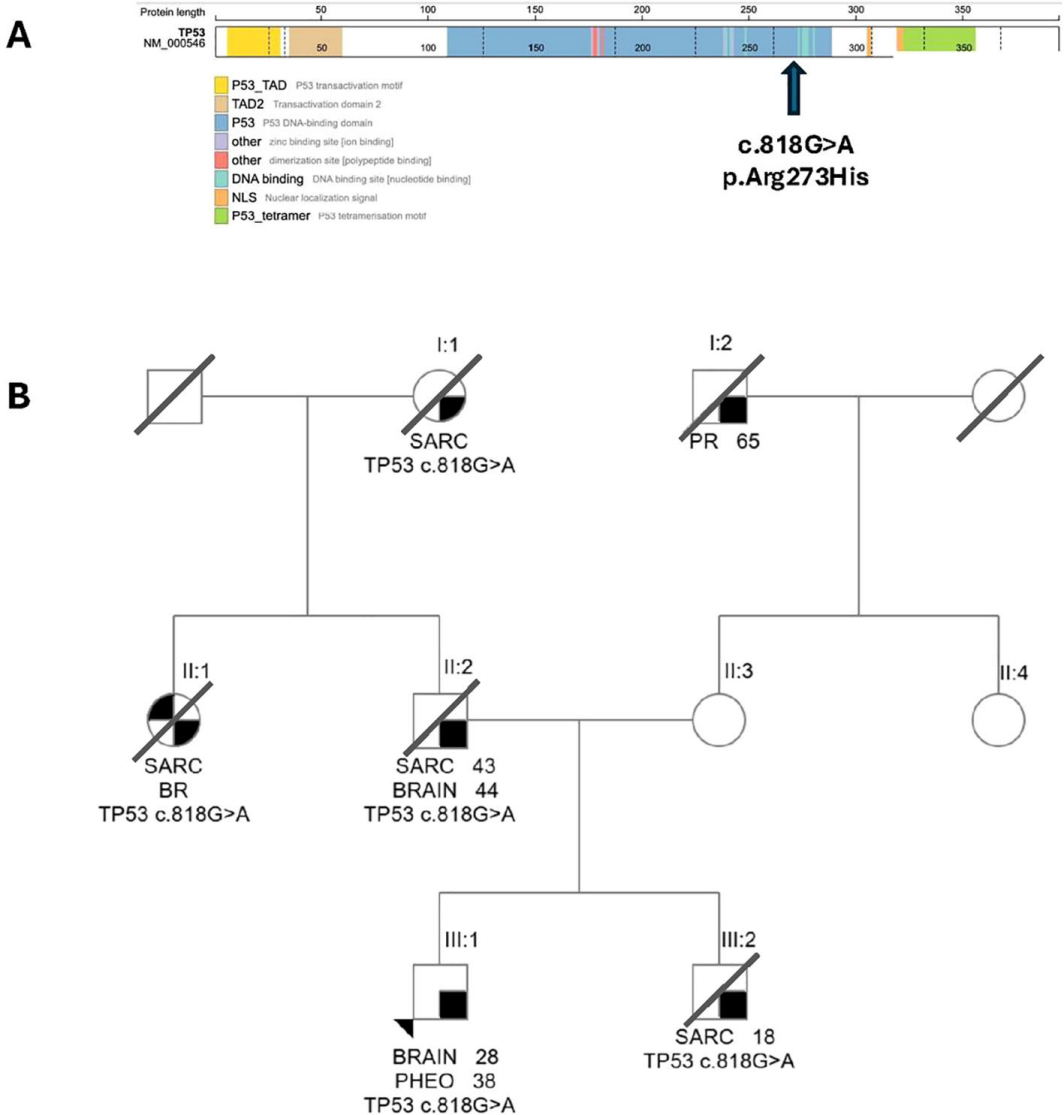
The TP53 germline variant to initialize. **(A)** Graphical representation of the known germline variant *TP53* c.818G>A; p.Arg273His. *TP53* c.818G>A; p.Arg273His causes an amino acid substitution within the DNA binding domain of the TP53 protein (transcript NM_000546.6). The graph is adapted from PeCanPIE (10). **(B)** Family cancer pedigree. Individuals within the familial unit are distinguished by Roman (denotes generation) and Arabic numerals (denotes individuals within each generation) positioned beneath a designated symbol (□ = male, ○ = female). Our patient is individual III:1 (black arrowhead). The tumor type is indicated for each individual (BRAIN = brain malignancy, BR = breast cancer; PHEO = pheochromocytoma; SARC = sarcoma; PR = prostate cancer), followed by age at diagnosis. Family members with *TP53* c.818G>A; p.Arg273His are denoted.

The patient is known to carry a germline *TP53* NM_000546.6: c.818G>A; p.Arg273His pathogenic variant (**Figure 2A**). This is a well-known pathogenic variant associated with LFS and has been reported in the germline of 139 patients accessioned in The *TP53* Database (R20, July 2019: https://tp53.isb-cgc.org), with 76 patients fulfilling either the classic Li–Fraumeni syndrome criteria or the Chompret criteria (11). This variant occurs in a recognized *TP53* hotspot codon within the DNA-binding domain (12). The variant displays non-functional transcriptional activity across eight different promoters, as assessed in yeast assays (13, 14), and there is also experimental evidence supporting a dominant-negative effect on wild-type TP53 in some cellular contexts (15, 16). The variant is present in gnomAD v2.1.1 in four individuals, exhibiting a minor allele frequency of 0.001593% [Genome Aggregation Database (gnomAD); accessed on 04/07/2024 from https://registry.opendata.aws/broad-gnomad]. The TP53 Variant Curation Expert Panel has thoroughly reviewed this variant and classified it as “pathogenic” according to the ACMG/AMP guidelines for variant interpretation (17).

The patient has a significant family history of various cancers consistent with LFS and inherited from the paternal side (**Figure 2B**), including a younger brother (patient III:2) who died of sarcoma at age 20, the father (patient II:2) who died of sarcoma at age 48, and both a paternal aunt (patient II:1) and grandmother (patient I:1) who died of sarcoma. The paternal aunt additionally had a history of breast cancer, further supporting LFS. Importantly, all of these family members were known carriers of the *TP53* NM_000546.6: c.818G>A; p.Arg273His pathogenic variant.

### TNWES of the pheochromocytoma and germline study

**Figure 3:**
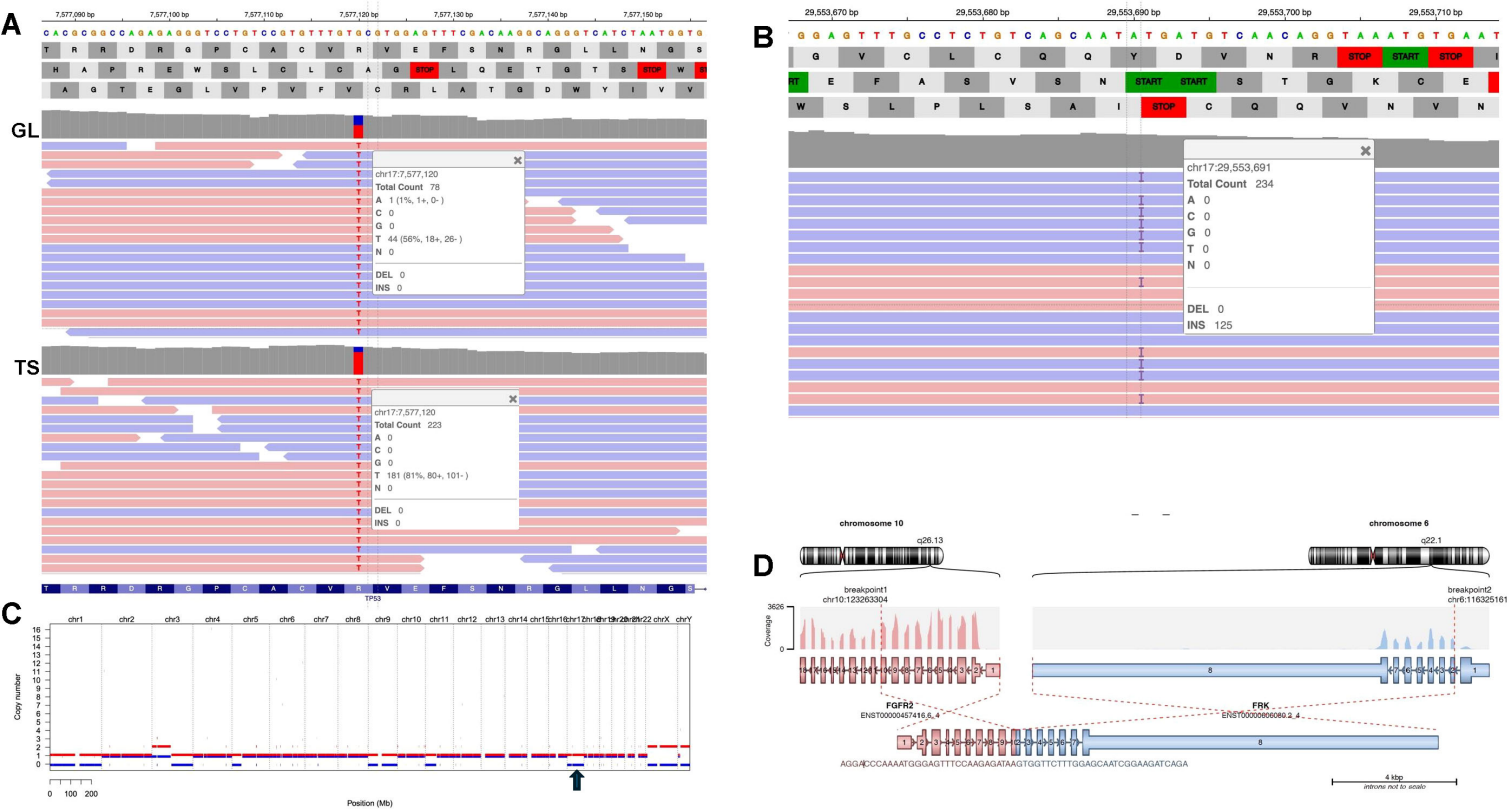
Tumor normal whole exome sequencing (TNWES) of patient pheochromocytoma. **(A)** IGV views of *TP53* c.818G>A; p.Arg273His variant detected in both patient blood and pheochromocytoma samples. The upper panel labeled GL is from the germline sequence (insert shows variant at 56%); the lower panel labeled TS is from the tumor sample (insert shows variant at 81%). (The *TP53* gene is on the reverse strand; therefore, the nucleotide change is identified as T instead of A. **(B)** IGV view of *NF1* c.2240dupT; p.Met747fs* variant detected in the patient’s pheochromocytoma sample. **(C)** Copy number analysis revealed a copy loss in both *TP53* and *NF1*, located on chr17p and chr17q, respectively. The arrow indicates the loss of one entire chr17. Colored bars indicate the two alleles; red bars above copy number 1 (*y*-axis) indicate gains; blue bars below 1 (*y*-axis) indicate loss. **(D)** *FGFR2::FRK*; NM_000141.5–NM_002031.3; chr10:123263304–chr6:116325161 detected in a patient’s bile duct adenoma. *FGFR2* exon 10 is joined in-frame to *FRK* exon 7.

The peripheral blood germline sample revealed the known pathogenic *TP53* p.Arg273His variant at a variant allele frequency (VAF) of 56% (**Figure 3A**) and two additional germline variants of uncertain significance in *ANKRD26* and *SBDS*. Somatic variants detected in pheochromocytoma included a likely pathogenic variant in *NF1* c.2240dupT; p.Met747fs* (**Figure 3B**) as well as numerous variants of uncertain clinical significance. Somatic copy number analysis revealed copy loss in *TP53* and *NF1*, likely due to loss of one entire chr17 (**Figure 3C**). Consistent with the detected *TP53* copy loss, the VAF of the germline *TP53* variant in the tumor was increased to 81% due to copy number loss.

### TNWES of the bile duct adenoma

Somatic analysis of the BDA revealed a likely pathogenic *FGFR2::FRK* fusion (NM_000141.5–NM_002031.3; chr10:123263304–chr6:116325161) joining exon 10 of *FGFR2* to exon 2 of *FRK* (**Figure 3D**). Conventional *FGFR2* fusions, typically associated with cholangiocarcinoma, retain the *FGFR2* kinase domain (11). In contrast, the detected fusion disrupts *FGFR2* prior to exon 17, which is predicted to result in loss of the encoded C-terminal portion of the tyrosine kinase domain. Notably, the breakpoint in *FRK* occurs at exon 2, which results in the retention of the FRK kinase domain that is structurally similar to other reported FRK activating fusions (18). Of interest, although copy number analysis was not interpretable due to the highly degraded DNA of this sample, the VAF of the *TP53* germline variant was 48%, suggesting the absence of LOH in this BDA.

## Discussion

We report the first case of pheochromocytoma with documented molecular evidence of biallelic inactivation of *TP53* in LFS, thereby implicating *TP53* in the pathogenesis of this cancer. Several cases of pheochromocytoma have been reported in patients with LFS (7, 19–21). The first case was reported in a patient from China with a strong family history of multiple cancers associated with LFS and characterized by a germline *TP53* p.Gly244Ser pathogenic variant (20). Unfortunately, that pheochromocytoma was not sequenced to assess the presence of somatic *TP53* variants that would have confirmed *TP53* in the pathogenesis of this tumor. This case is included in the germline section of The *TP53* Database as the only reported pheochromocytoma in their series of 4,450 germline *TP53* pathogenic variants (22). Another case occurred in a patient with highly aggressive metastatic pheochromocytoma who was reported to have both germline *TP53* (p.Arg248Gln) and *SDHB* (p.Arg46*) pathogenic variants (21), confounding the role of the *TP53* germline variant, as *SDHB* pathogenic germline variants are well-established contributors to the development of pheochromocytomas (23). As with the previous case, neither chr17p LOH nor a second somatic inactivating *TP53* pathogenic variant was reported for this patient’s pheochromocytoma.

*TP53* is perhaps the most well-known of the tumor suppressor genes (TSGs), with alterations occurring in approximately 40% of human cancers and spanning many different cancer types and age groups. As a general rule, TSGs demonstrate inactivation of both alleles either through a second inactivating pathogenic variant within the alternate allele of the *TP53* gene or more commonly through LOH involving the entire alternate *TP53* locus (24). Most LOH events are caused by local or arm-level deletions that include the entire second *TP53* allele, frequently identified by routine cytogenetic analysis or conventional LOH assays. However, other inactivating alterations have been reported to occur, including copy-neutral LOH, *TP53* promoter methylation, and whole chr17 loss (25). Notably, in the current case, whole chromosome 17 loss resulting in *TP53* LOH was detected in this aggressive pheochromocytoma.

Despite its prevalence in cancer, *TP53* pathogenic variants are rare in pheochromocytoma, found in as low as 0.6% of both inherited and sporadic cases (26). The *TP53* Database includes 29,346 somatic entries of various cancers and 4,450 germline entries (21). There is only one pheochromocytoma represented in the germline variants as mentioned above, and there are no entries under the somatic variants. The TCGA cohort of 173 cases of pheochromocytoma/paraganglioma (PCC/PGL) reports only one case with a pathogenic *TP53* variant (6). It is unclear as to why *TP53* pathogenic variants are so underrepresented in pheochromocytomas. However, the low incidence of *TP53* pathogenic variants in sporadic pheochromocytoma may in part explain the rare occurrence of pheochromocytoma in LFS.

On the other hand, *NF1* pathogenic variants have been reported in up to 26% of cases of sporadic PCC/PGL and for germline pathogenic variants in up to 7.7%–14% of familial cases (27, 28). Inactivating alterations of *NF1* are oncogenic due to loss of its ability to activate RAS GTPase activity, which leads to upregulation of the RAS/MAPK pathway (27). As such, *NF1* behaves as a tumor suppressor gene and has been implicated in numerous different cancer types, most commonly in neurofibromas and melanoma, but also in breast cancer, ovarian cancer, colon cancer, glioblastoma, PCC/PGL, and many others. In the TCGA PCC/PGL cohort, there is no observed co-occurrence of *TP53* and *NF1* variants, although 12% of PCC/PGL cases in TCGA exhibit *NF1* variants.

**Figure 4:**
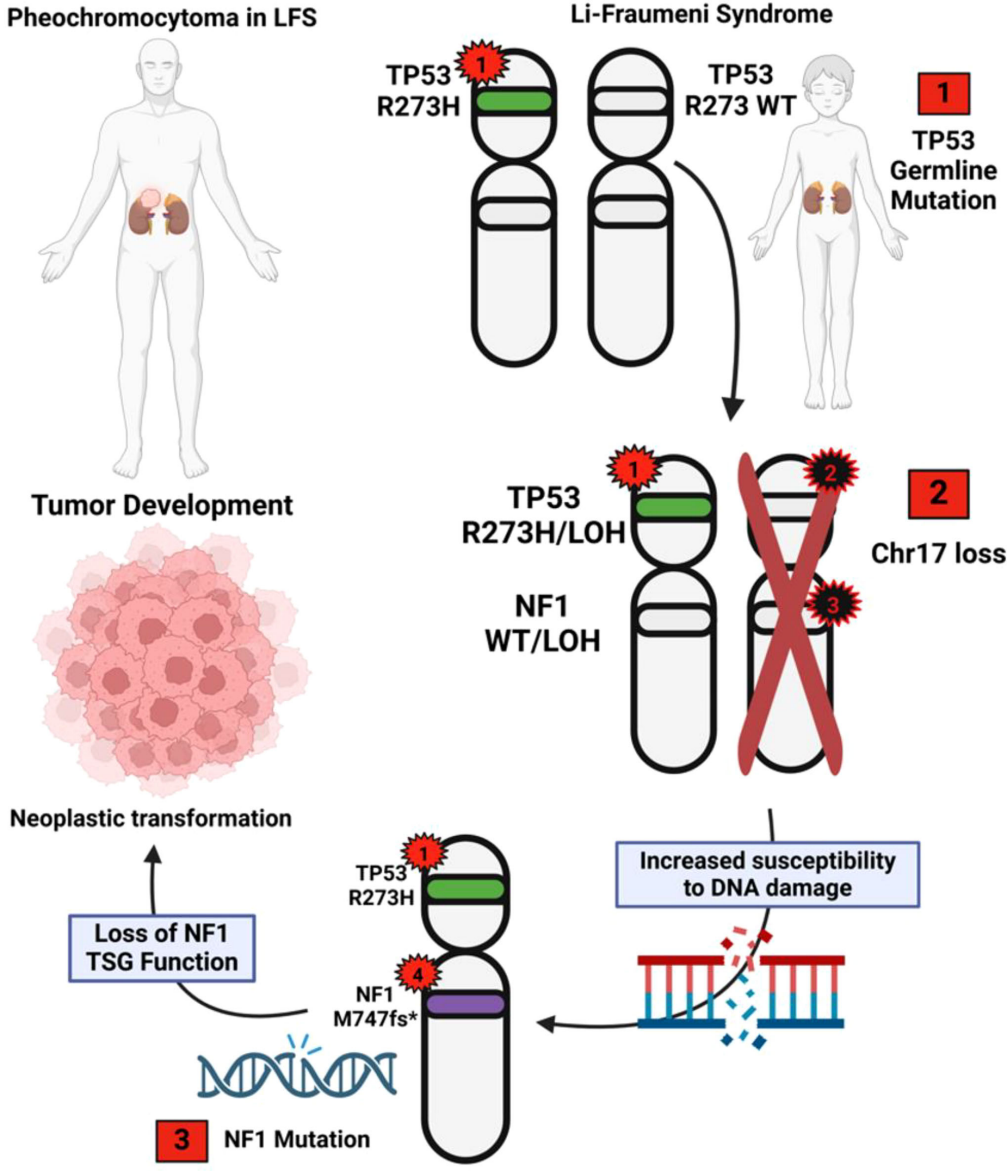
The proposed 3-event, 4-hit model of the genetic chronology of this tumor. 1) Germline *TP53* (hit 1) pathogenic variant; 2) loss of chr17 -> *TP53* LOH (hit 2) and *NF1* LOH (hit 3); 3) *NF1* somatic pathogenic variant (hit 4). Red stars along chromosome 17 represent pathogenic small nucleotide variants in *TP53* or *NF1*; black stars represent copy loss variants. Numbers within stars indicate hit order (hits 2 and 3 are presumably simultaneous). The green bar on chromosome 17p represents the germline *TP53* variant; the purple bar represents the somatic *NF1* variant on chromosome 17q. Clear bars represent the associated wild-type alleles. Red boxes indicate the three proposed events accounting for four genetic alterations (hits) involved in pheochromocytoma pathogenesis.

The rare co-occurrence of these two mutated tumor suppressor genes in pheochromocytoma coupled with the loss of an entire chr17 in our case leads us to propose the following 3-event, 4-hit model of pathogenesis for the current tumor (**Figure 4**). The initial event (event 1) is the germline *TP53* pathogenic variant, representing the first mutational “hit” (hit 1). While this variant has been shown to have some ability to suppress normal functioning WT-*TP53* in some experimental systems (dominant-negative effect), most variants showing dominant negative effects (including the p.Arg273His) do not appear to be sufficient for tumorigenesis, and the vast majority of tumors show inactivation of the second allele (15, 16, 25, 29, 30). Thus, loss of one copy of chr17 represents the second event (event 2) and results in hemizygous loss of all genes on chr17 including *TP53* (hit 2 by LOH) and *NF1* (hit 3 by LOH). Biallelic loss of a functional TP53 checkpoint puts the neoplastic cells at risk for subsequent pathogenic variant events that cannot be effectively repaired, and the hemizygous state of *NF1* puts them at a higher risk for a pathogenic variant inactivating event. Finally, the second allele of *NF1* undergoes an inactivating pathogenic variant (event 3) (hit 4), setting up the condition for pheochromocytoma development. A similar 3-event, 4-hit mechanism resulting in biallelic inactivation of two TSGs on the same chromosome has been proposed for the pathogenesis of schwannomatosis, a tumor predisposition syndrome in which the germline *SMARCB1* pathogenic variants show large chromosomal deletions on chr22q that not only result in *SMARCB1* LOH but also render the nearby *NF2* gene hemizygous. This increases the chance of *NF2* inactivation by a second pathogenic mutational event, which is the molecular profile of many of the schwannomas that develop in this germline tumor predisposition syndrome (31).

In summary, to our knowledge, this is the first report of pheochromocytoma in an LFS patient demonstrating biallelic inactivation of *TP53*, thereby implicating *TP53* in its pathogenesis. We have proposed a multistep model in which the *TP53* loss serves as a foundational, permissive driver that enables genomic instability and clonal evolution, whereas *NF1* loss constitutes a lineage-defining oncogenic driver directly responsible for pheochromocytoma tumor initiation and progression. We hypothesize that the mechanism of *TP53* second allele inactivation and loss of the entire chromosome 17, which included one *NF1* allele, facilitated the possibility of biallelic inactivation of *NF1* and led to this rare case of pheochromocytoma. This case underscores the importance of comprehensive genetic and functional characterization of tumorigenesis in atypical presentations of cancer predisposition syndromes with broad tumor spectra, enabling the elucidation of context-specific pathogenic mechanisms and refinement of genotype–phenotype correlations relevant to precision surveillance and care.

## Methods

### Patient enrollment

The patient participated in three clinical trials conducted at the National Cancer Institute and *Eunice Kennedy Shriver* National Institute of Child Health and Human Development, focusing on investigations related to LFS, pheochromocytoma, and molecular analysis of endocrine tumors (Clinical Trial Numbers: 11-C-0255, 09-C-0242, 00-CH-0093). All three protocols received approval from the Institutional Review Board of either the National Cancer Institute or the National Institute of Child Health and Human Development. All experiments were conducted in accordance with relevant guidelines and regulations, and informed consent was obtained from all participants prior to their involvement in the study.

### Germline whole exome sequencing

Genomic DNA was extracted from peripheral blood using QIAsymphony DSP DNA Midi Kit (Qiagen, Redwood City, CA) and quantified with a Qubit fluorometer (Thermo Fisher Scientific, Waltham, MA). Whole exome DNA sequencing library was prepared with 100 ng of tumor or normal DNA using the Nextera Flex enrichment kit with the exome probe panel (Illumina Inc., San Diego, USA). The final enriched libraries were pooled and loaded for the target coverage of 50× for normal DNA and 150× for the tumor DNA and sequenced on a NovaSeq 6000 (Illumina Inc.). The Comprehensive Oncologic Molecular Pathology and Sequencing Service (COMPASS) Exome Germline Sequencing assay employed next-generation sequencing to detect germline variants in human genome coding regions. Data analysis utilized the COMPASS Somatic/Germline Bioinformatic Pipeline (see Bioinformatics section below), and variants were classified using QIAGEN Clinical Insight (QCI) Interpret software. Pathogenic and likely pathogenic variants were reported; final interpretation considered variant pathogenicity, frequency, and supporting literature. A board-certified pathologist performed the final review and generated the report adhering to HUGO Gene Nomenclature Committee (HGNC) and Human Genome Variation Society (HGVS) guidelines.

### Somatic whole exome sequencing

Tumor DNA and total RNA were extracted from formalin-fixed paraffin-embedded tissue sections using FFPE tissue kits from Qiagen. The COMPASS Exome Somatic Sequencing test, based on the Nextera Exome Kit, detects somatic pathogenic variants in human genome coding regions. Data analysis employed the COMPASS Somatic/Germline Bioinformatic Pipeline, and variants were classified using QCI Interpret. Pathogenic, likely pathogenic, and variants of uncertain significance were reported, with tiered actionability classifications. Copy number estimation was assessed using the Sequenza package v3.0.0, which was integrated into the custom pipeline described in the Bioinformatics section below. Sequenza uses paired tumor-normal DNA sequencing data to estimate tumor cellularity and ploidy, and calculates allele-specific copy number profiles (32).

### Bioinformatics analysis pipeline

Whole exome sequencing data were processed using a custom in-house NGS bioinformatics pipeline (v5.0.0) to report both somatic and germline mutations including single-nucleotide variants (SNVs) and small indels (1–20 bp) in tumor and germline samples. The raw files (bcl files) from the sequencer were converted to fastq files using the Illumina-provided bcl2fastq (v2.20.0) tool. Prealignment QC was performed using FastQC (v0.11.2). The fastq files were mapped to the hg19 build of the human genome using BWA-mem (v0.7.17). Duplicate reads were removed using Picard’s MarkDuplicate command (v2.18.27), and bam files were processed using Genome Analysis Toolkit, GATK (v3.8-1) following the best practices provided by the Broad Institute. The final bam files were used to identify SNVs using Germline Haplotype (v3.8-1) caller and Somatic Mutect (v1.1.7) caller. For the detection of small indels (1–20 bp), STRELKA (v2.9.10) was used for calling somatic indels, and Haplotype Caller was used for germline indels. The complete code of the pipeline (v5.0.0) along with the configuration files, tool versions, etc. is available on the GitHub repository (https://github.com/manGitHub/ngs_pipeline_COMPASS_master). The same pipeline was used for RNA-seq data analysis, and the individual steps were described in the subsequent RNA analysis section. CNV estimation was performed using the Sequenza package (v3.0.0) implemented in the same in-house pipeline.

### RNA analysis and reporting

Exome RNA sequencing library was prepared with 100 ng of tumor RNA using the Illumina RNA Prep, Tagmentation (L) with Enrichment kit pairs with the same exome probe panel for DNA. The final enriched libraries were sequenced on NextSeq 550DX or NovaSeq 6000 (Illumina Inc.). A custom in-house pipeline (https://github.com/manGitHub/ngs_pipeline_COMPASS_master) processed RNA data and detected fusions using Arriba (v2.2.1). Sequences were compared against the human genome reference GRCh37/hg19, with gene annotation from Gencode GTF (v36lift37). The fusion file from a 600-gene panel was uploaded to QCI for filtering, annotation, classification, and interpretation. Fusions with a minimum of 10 supporting reads underwent clinical interpretation.

## Data Availability

Original datasets are available in a publicly accessible repository:This data can be found here: [https://www.ncbi.nlm.nih.gov/projects/gapprev/gap/cgi-bin/preview1.cgi?GAP_phs_code=lIpJswFFDLew6v7v/dbGaP:phs003861.v1.p1].
