# Supplementary material for: Case Report: Rare pheochromocytoma in a patient with Li–Fraumeni syndrome: a 3-event, 4-hit model of pathogenesis
Source: Front Oncol. 2026 Mar 11;16:1714565. doi: 10.3389/fonc.2026.1714565 (PMC13012990; doi:10.3389/fonc.2026.1714565)
Supplement: Supplementary file 1 [file DataSheet1.pdf]

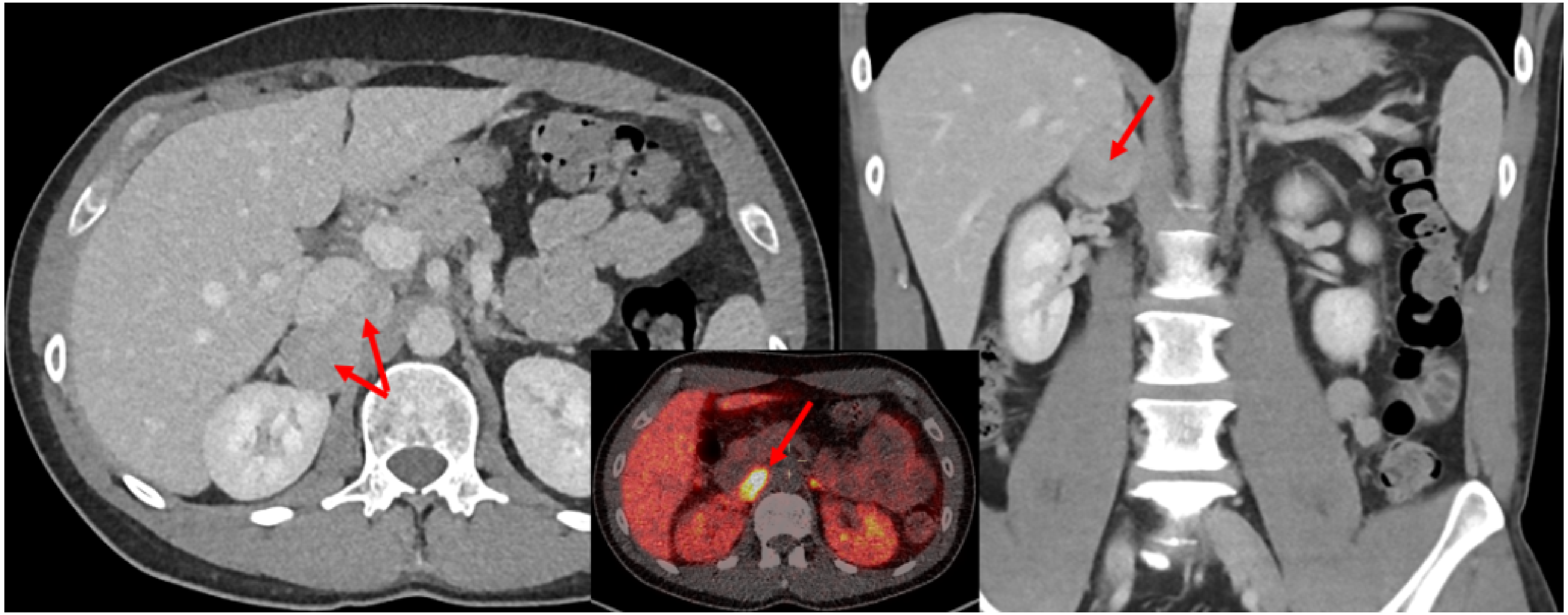

Supplemental Figure 1. Computed tomography (CT) scan of the patient. CT axial and coronal imaging demonstrated a bilobed, fairly homogenous mass (red arrows) measuring 5.5 x 3.2 cm in greatest dimensions with avidity (inset red arrow) on Ga-68 DOTATATE imaging (central inset image).
